# Management of primary anorectal mucosal melanoma during the COVID-19 pandemic

**DOI:** 10.3332/ecancer.2023.1610

**Published:** 2023-10-05

**Authors:** Edgar Fermín Yan-Quiroz, Folker Mijaíl Agreda-Castro, Lita Diaz-Lozano, Richard Tenazoa-Villalobos, Lissett Jeanette Fernández-Rodríguez

**Affiliations:** 1 Hospital de Alta Complejidad Virgen de la Puerta – EsSalud, La Esperanza 13013, Perú; 2 Faculty of Medicine, Universidad Privada Antenor Orrego, Trujillo 13008, Perú; 3 Hospital Víctor Lazarte Echegaray – EsSalud, Trujillo 13006, Perú; 4 Hospital Regional de Lambayeque – Ministerio de Salud, Chiclayo 14012, Perú; a https://orcid.org/0000-0002-9128-4760; b https://orcid.org/0000-0003-4057-6365; c https://orcid.org/0000-0003-2842-369X; d https://orcid.org/0000-0003-3622-9408; e https://orcid.org/0000-0002-4357-4261

**Keywords:** anorectal melanoma, nivolumab, COVID-19, surgery, radiation therapy, radiotherapy, Peru

## Abstract

Anorectal melanoma is a rare and difficult-to-diagnose highly malignant cancer with a poor prognosis. The treatment usually involves surgery and often includes adjuvants such as radiation therapy and immunotherapy. We present a case of a 77-year-old Peruvian who was eventually diagnosed with this cancer during the COVID-19 pandemic, which complicated her treatment and allowed the cancer to spread. Her treatment included abdominoperineal resection, bilateral pelvic lymphadenectomy, left internal iliac vein raffia and end colostomy, followed by 3D radiation therapy (50 Gy, 25 sessions) and systemic treatment with nivolumab, all of which were well tolerated. The patient was alive as of 20 August 2023, having survived for more than 3 years since the onset of symptoms.

## Background

Melanoma is a malignancy that originates in melanocytes. This cancer almost always appears on the skin as cutaneous melanoma but may also occur in the retina and mucosa, such as in the anorectum. Mucosal anorectal melanoma (MAM) is very rare, accounting for approximately 1% of all melanoma cases. It has an unfavourable prognosis due to difficult diagnosis, rarity, and malignancy. Currently, the median cause-specific survival rate is very poor compared to cutaneous melanoma: 33, 18 and 6 months for localised, regional, and distant disease, respectively [1–11].

Diagnosis of MAM is difficult because it is often asymptomatic or presents with non-specific symptoms such as rectal bleeding or pain, a feeling of mass in the anus, tenesmus, pruritus, weight loss, lower gastrointestinal bleeding and/or a change in bowel habits [8, 10, 12]. These symptoms are frequently seen in other more common diseases and conditions, such as haemorrhoids [7]. Correct diagnosis of MAM usually occurs after common conditions are eliminated and often requires rectoscopy, histopathology and auxiliary tests [1–10].

Furthermore, the staging, management and treatment of MAM remain a challenge because clinical outcomes do not match the extent of the disease at diagnosis in the same way as cutaneous melanoma [8, 11]. This may be because mucosal melanomas tend to have different genetic profiles and mutations than cutaneous melanomas. For example, BRAF-activating mutations are less common in mucosal melanoma than in cutaneous melanoma, while other mutations, such as KRAS and cKIT, appear to be more common, making the latter two important in the pathogenesis and targeted treatment of MAM [13, 14]. Therefore, it is necessary to develop a separate staging system, which is complicated by the rarity of cases [11]. Despite this, staging systems have been proposed for mucosal melanoma, but without distinction with respect to the location of the primary tumour [4, 11, 15].

The rarity and malignancy of MAM make an early multidisciplinary approach in a well-resourced clinic important to improve prognosis. Because treatment recommendations are based on small studies or studies that include different tumour locations, the experience of the oncologist, the extent or recurrence of the disease and the wishes of the patient should be considered in treatment. Treatment of MAM usually involves surgery. However, there is less evidence on whether the type of surgery and adjuvant treatment, such as targeted therapy, chemotherapy, immunotherapy or radiotherapy (RT) relate to improved overall survival [5, 11, 16–19].

We present a case of a woman diagnosed with MAM that highlights the importance of communication, rapid medical attention and prompt diagnosis as a part of the initial patient assessment. Because treatment took place during the recent COVID-19 pandemic, certain procedures were delayed due to government-related restrictions [20] that may have jeopardised the patient.

## Patient information

In November 2019, a 77-year-old Peruvian woman was seen in the Gastroenterology Department of the EsSalud Hospital in Chimbote, Peru. The patient had an unremarkable history, except that she had been treated for thyroid cancer 10 years prior with surgery and ^131^I. Furthermore, the patient had not previously been diagnosed with any other form of melanoma. At this visit, her main complaint was anal pruritus.

A rectoscopy was performed, where an elevated polypoid lesion of 18 mm in diameter with a multinodular erythematous surface and an adherent clot was observed in the distal rectum. A wire loop polypectomy was performed to remove the lesion. Macroscopic examination of the biopsy revealed a fragment of tissue with a polypoid appearance and a brownish surface. Microscopic examination revealed a poorly differentiated malignancy, suggesting carcinoma or melanoma.

The patient went to follow-up 31 days after rectoscopy where she was informed of her histopathological result. The patient reported daily bowel movements without bleeding, occasional mild anorectal pain and episodes of gas and nausea. Abdominal and pelvic tomography and chest radiography revealed mild diffuse liver disease, interstitial lung disease and aortic atheromatosis likely associated with age. A thickening of the rectal area was also observed. Blood analysis was apparently normal, although prothrombin time was slightly elevated.

Five additional subsequent follow-ups, some of which were virtual, were conducted between January and November 2020 by the same service in Chimbote. During these follow-ups, the patient complained of dyspepsia and was prescribed proton pump inhibitors. In October, she complained of traces of blood in her stool during a telephone consultation. The patient was prescribed intrarectal mesalazine daily for 5 days and anti-haemorrhoidal cream. This partially fixed the complaint. In November 2020, rectoscopy follow-up was indicated but was not completed due to local COVID-19 pandemic regulations [20].

The patient was seen for rectoscopy in March 2021 at Víctor Lazarte Echegaray Hospital in Trujillo. Above the dentate line, a friable and poorly defined tumour with a necrotic surface and areas of haemorrhage, irregular edges and clots adhered to its surface was evident. The tumour measured approximately 30 mm and obstructed 30% of the rectal lumen, extending 5–10 cm from the margin of the anal mucosa. Multiple biopsies were taken, which caused bleeding. Histopathology indicated a poorly differentiated malignancy with positive vimentin, positive melan-A and focal positive Hmb45 markers. Biopsies were negative for pankeratin, leukocyte common antigen, CD 56 and ki67: 60% tumour markers. Histomorphology and immunohistochemistry were compatible with malignant melanoma.

The patient also underwent contrast-enhanced tomography of the thorax, abdomen and pelvis. The most important observation was nodular thickening of the posterior wall of the rectum measuring 10 × 5 mm associated with two small adjacent nodes in the perirectal fat. No suspicious osteolytic or blastic lesions were observed. These results lead to the diagnosis of localised primary anal canal melanoma.

In April 2021, the patient was evaluated by the oncology service, which transferred the patient to oncological surgery. The patient was informed of her diagnosis and that the lesion is extensive and has recurred. After consultation, the patient consented to surgery to remove the tumour. In May 2021, the patient underwent exploratory laparotomy, abdominoperineal resection (APR), bilateral pelvic lymphadenectomy, left internal iliac vein raffia, one Blake drain placement and end colostomy.

Operative findings included the sigmoid colon attached to the pelvic cavity, no evidence of suspicious adenopathies at the level of the inferior mesenteric artery or in the internal or external iliac vessels, no palpable tumour or abnormally thick tissue at the level of the rectum and no lesions observed at the level of the anal canal. A soft, flat, blackish lesion of 4 cm was observed on the posterior face of the lower rectum. There were no signs of intra-abdominal free fluid, organ lesions, carcinomatosis or distant metastases. The patient developed favourably and was discharged a week later. Genetic tests were not performed on the tumour as they were not available at the hospital where the patient was treated.

**Figure 1.**
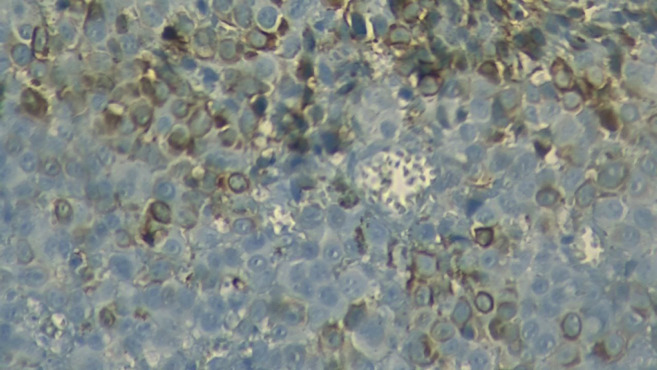
Micrograph of the unstained tumour. Micrograph (40×) of the tumour excised during surgery; the brown colouration of some cells reveals the presence of melanin, suggesting melanoma.

**Figure 2.**
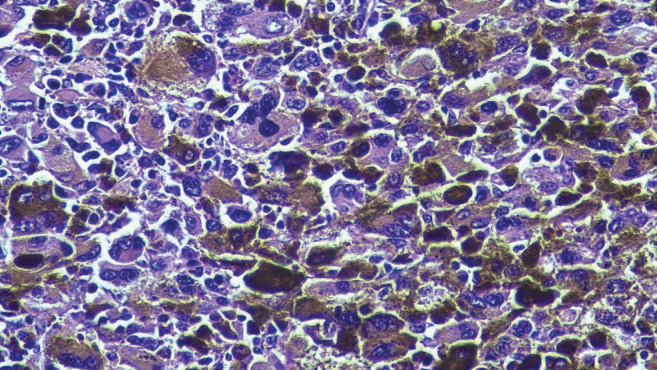
Micrograph of the tumour stained with haematoxylin-eosin. Micrograph (40×) of the tumour excised during surgery with haematoxylin-eosin stain. The anorectal mucosa is infiltrated by malignant epithelioid neoplastic cells with prominent nucleoli and melanin.

The postoperative histopathological result revealed pigmented and ulcerated anorectal invasive melanoma, with the presence of pleomorphic giant cells (Figures 1 and 2). The predominant cell type was epithelioid. Additional observations were that the dissemination phase was radial and vertical with mitosis of 3/mm^2^. The tumour infiltrated up to the muscularis propria, with a depth of 5 mm. Neither vascular-lymphatic invasion nor perineural invasion with nonenergetic positive tumour lymphocytic infiltrate was observed. The anal (0.5 cm) and radial (0.7 cm) surgical margins were free of neoplasia. Four out of 17 regional lymph nodes were affected, but neither the 12 resected right omental lymph nodes nor the 5 resected left omental lymph nodes were affected.

**Figure 3.**
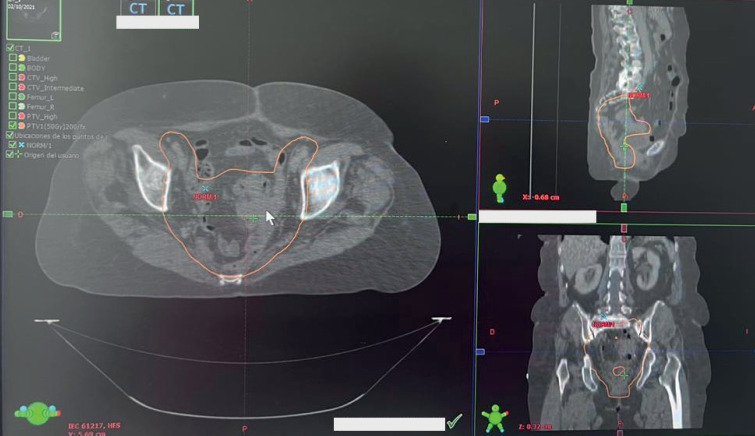
RT planning target volume. Planning target volume of the postoperative patient is outlined in orange. The large image is along the transverse plane, whereas the top and bottom images are of the sagittal and coronal planes, respectively.

**Figure 4.**
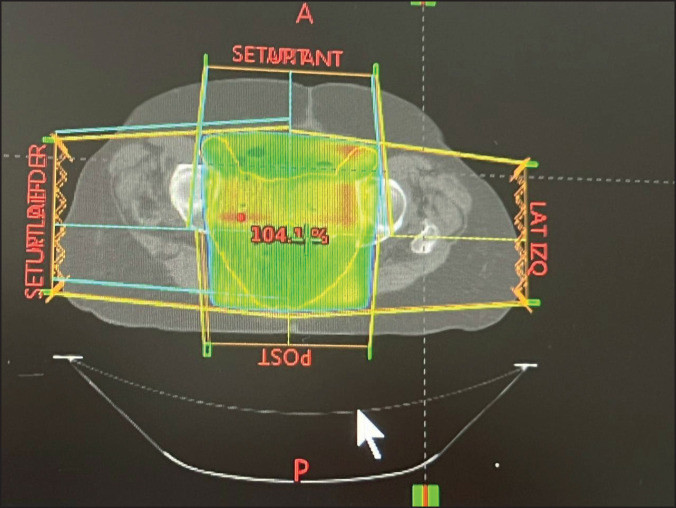
Radiation planning dose. 3D pelvic cavity RT planning in the postoperative patient at a dose of 50 Gy in 25 fractions. This transverse image presents the dose distribution to the pelvic cavity.

The patient was evaluated by medical oncology which referred her to the radiotherapy service for a dose of 50 Gy in 25 3D sessions to the pelvic region using a CLINAC 2300 model IX linear accelerator (Varian Medical Systems, Inc., USA), which was adequately tolerated (Figures 3 and 4). The last session was held in November 2021.

In August 2022, the patient was operated on to correct a hernia that developed from the colostomy. In November 2022, tomography and magnetic resonance imaging of the abdominopelvic region suggested peritoneal carcinomatosis. The diagnosis was confirmed in March 2023. Two months later, the patient began to receive 240 mg of nivolumab every 2 weeks. The patient is tolerating the colostomy bag and nivolumab. As of August 2023, the patient is still alive and has completed six rounds of nivolumab. So far, she has lived 46 months since the onset of symptoms.

## Discussion

Although both MAM and cutaneous melanomas involve melanocytes, each disease has distinct characteristics. Therefore, it is better to consider MAM as a separate form of cancer with distinct causes, staging and treatment. Developing evidence-based methods for staging and treatment is difficult due to the rarity of cases, although progress has been made.

Immunosuppression and oxidative stress in the anal region have been proposed as causes of MAM, but more research is needed to clearly determine the mechanism of pathogenesis. It has been proposed that MAM originates from melanoblasts, amine precursor uptake and decarboxylation (APUD) cells or Schwannian neuroblastic cells [3, 11]. Most MAM tumours develop the anal canal or pectinate line and are often pigmented. These tumours are usually 2.9–3.8 cm in diameter and may appear to be ulcerated, flat or even polypoid [4]. The median age at the time of MAM diagnosis is approximately 70 years [9].

The medical history of our patient also led us to consider whether previous cancer or cancer treatment may increase the risk of MAM. Three case reports showed relevant results: diagnosis of MAM after breast cancer treatment [3], metachronous thyroid cancer and MAM [21] and concomitant uveal melanoma and thyroid cancer [22]. In addition, a history of thyroid cancer appears to increase the risk of cutaneous melanoma and vice versa [23, 24]. Given this information, it is possible that a medical history of cancer, especially thyroid cancer, predisposes patients to MAM or vice versa.

There is no commonly accepted guide for staging MAM due to its rarity, but some guidelines for related cancers have been used in the literature. A traditional four-stage model was proposed: Stage I, no infiltration of the muscle layer; Stage II, muscle infiltration; Stage III locoregional lymphadenopathy and Stage IV distant metastasis [4]. According to this proposal, the patient had Stage III MAM at the time of colostomy. A more recent TNM-based staging system was also proposed, suggesting T2N2M0 and a IIIB stage based on invasion of the muscularis propria, the four lymph nodes involved, and no evidence of brain, liver or lung metastases at the time of colostomy, giving a median survival of 2.1 years [11, 15]. Using lactate dehydrogenase (LDH) concentration to stage vulvo-vaginal mucosal melanoma [25] and cutaneous melanoma [26] is recommended, and one proposed system for MAM mentions its use to determine median survival for Stage IV patients [11]. LDH levels could indicate the burden of the disease as with cutaneous melanoma. The LDH blood concentration was not measured in our patient, since the stage was determined to be Stage III and there was no research that correlated LDH with survival in MAM [27].

Like staging, MAM treatment has not been standardised due to the lack of strong evidence to support a particular approach [6]. However, treatment for MAM consists of three broad approaches that reflect general cancer treatment: surgery, RT and chemotherapy [11]. These treatment approaches can be used separately or together. Because of this lack of standardisation, the treatment plan for a patient with MAM should include expert opinion, patient desires, disease stage and local realities and expertise.

Historically, surgery has been the primary treatment for MAM [19], but the necessity of surgical treatment has been questioned [28], as it does not appear to affect overall survival [11, 29]. Two surgeries are commonly used to excise the tumour with sufficient margins: APR and wide local excision (WLE). There is disagreement between which procedure is best [5, 8, 16–19, 30–33]. The more radical APR approach increases chances of clean surgical margins, but involves a larger amount of anatomy, making it a riskier procedure. APR also results in a permanent colostomy and risks sexual and urological problems. On the other hand, WLE holds the promise of preserving more anatomy but is generally indicated for small lesions less than 1 mm thick [4]. The difference in failure and recurrence rate between the two procedures is minor at best; some studies do not show a significant advantage between the two [5, 8, 16–19, 30–33]. In addition, less radical endoscopic mucosal resection has been described for less advanced cases [34].

Resection of lymph nodes is controversial, as it can increase complications [34]. The mesorectal, pelvic sidewall and inguinal lymph nodes are the most likely to be involved [29], but it is unclear whether the involvement or removal of these nodes improves the prognosis. Therefore, some recommend lymphadenectomy only for palpable or regional nodal disease [34].

Imaging studies performed before surgery did not reveal the complete depth of the tumour to the muscularis propria, which was only evident during surgery. Other surgical observations indicated that the tumour was localised and did not appear to have spread. Given these observations, our desire to quickly achieve local control, local surgical expertise, patient preference and tumour extent and recurrence, we chose APR with lymph node excision because the lesion was extensive, had infiltration greater than 4 mm [4], had poorly defined edges and had recurred. This decision has precedent in the literature, as a Stage IIIA case achieved good local control with similar surgery [34]. We recognise that this procedure has lifelong consequences for the patient, but we felt that this choice was best because it eliminated the primary tumour.

Combining surgery with adjuvant immunotherapy or RT to treat MAM has also been questioned, and apparently offers no survival benefit, with several studies failing to show statistical significance [11]. However, RT may decrease the probability of recurrence or improve local control, especially when there is lymph involvement [5, 11]. Combining surgery with extended field or dose-limited hypofractionated RT at doses of 25–36 Gy in 5–6 fractions can also decrease the likelihood of recurrence. Good control (80%+) and preservation of anatomy have been observed, but the overall 5-year survival rate for MAM remains at approximately 30% [35].

In our case, we modified the RT dose to 50 Gy in 25 sessions using the 3D technique and conventional fractionation (Figures 3 and 4) [36, 37]. Although this procedure is likely suboptimal from the point of view of treatment, control, and collateral damage, a large volume of patients required the use of a more time-efficient technique [38]; the patient tolerated the treatment adequately.

Adjuvant systemic therapy has also been recommended for resected MAM; its efficacy is currently the subject of ongoing clinical trials (for example, NCT04462965, NCT04180995, NCT04472806, NCT03241186 and NCT05436990). Most of the agents studied are immune checkpoint inhibitors and immunomodulators like interferon, although small molecules such as cisplatin, temozolomide, cabozantinib and dacarbazine/temozolomide have been or are being studied [11, 39].

Programmed cell death-1 (PD-1) immune checkpoint inhibitors down-regulate the PD-1 receptor. PD-1 is present on the surface of several immunocytes; activation downregulates immune response. Cancer cells, including melanoma, express PD-L1 and PD-L2 ligands that activate PD-1, allowing them to evade the immune response. However, by inhibiting the inhibitor, PD-1 immune checkpoint inhibitors deactivate this cancer cell countermeasure and theoretically restore the immune response [40]. Immune checkpoint inhibitor therapy for mucosal melanoma appears to improve survival in some cases, but not all studies have shown a statistically significant improvement in disease-specific and overall survival. Therefore, it is not yet recommended to abandon surgery for immune checkpoint therapy [41]. An additional area of research is the synergistic effect of RT and immune checkpoint therapy, which may further support the use of RT, even if adjuvant RT may not improve survival [42].

Nivolumab is a whole-antibody PD-1 inhibitor approved for several types of cancer, including cutaneous melanoma, where it has been shown to improve recurrence-free survival [43, 44]. The combination of nivolumab with other checkpoint inhibitors or small molecules is currently being investigated with some promising initial results [45]. However, nivolumab and related inhibitors have side effects associated with an overactive immune system [40, 46–48], making it necessary to carefully monitor the patient, but the undesirable side effects of nivolumab appear to be less than other agents or in combination with other systemic agents [43, 49]. In our case, the patient has tolerated six rounds of nivolumab.

An unfortunate consequence of the recent COVID-19 pandemic is that rectoscopies were delayed, which probably allowed the cancer to spread, and inhibited rapid multidisciplinary management of the neoplasm [20]. This delay had a negative impact on patient treatment options and allowed the recurrent tumour to spread to the point where complete remission was unlikely. Experience from the recent pandemic supports keeping cancer treatment centres open during pandemics, following infection reduction precautions and prudently postponing only those treatments with uncertain benefits [50, 51].

## Conclusion

Early detection and prompt appropriate treatment are key to increasing cancer survival. These are contingent on proper screening, evidence-based treatment plans and a competent and well-supplied health care system. This case is illustrative of how gaps in this system, especially those caused by the recent pandemic and high patient volumes, can result in undesirable treatment outcomes, especially for rare cancers with poor prognosis, and undefined staging and treatment. One wonders if rectoscopies and surgeries had not been delayed, carcinomatosis would not have developed as soon as it did in this case. Despite these difficulties, we present a case of MAM treated favourably with APR, RT and nivolumab.

## Conflicts of interest

The authors declare that they have no economic or non-economic conflicts of interest in the publication of this article.

## Funding statement

This project was self-funded by the authors and did not receive funding from any outside source.

## Author contributions

Edgar Fermín Yan-Quiroz: Patient care and manuscript writing.

Folker Mijaíl Agreda-Castro: Patient care and writing the introduction.

Lita Diaz Lozano: Review of pathological data.

Richard Tenazoa Villalobos: Collection of medical history data.

Lissett Jeanette Fernández-Rodríguez: Writing and editing of the manuscript.

## Ethical considerations

The patient consented to the publication of her case and related images.
